# Acceleration of emergence of *E. coli* antibiotic resistance in a simulated sublethal concentration of copper and tetracycline co-contaminated environment

**DOI:** 10.1186/s13568-020-01173-6

**Published:** 2021-01-07

**Authors:** Jinmei Li, Irfan Ali Phulpoto, Guilong Zhang, Zhisheng Yu

**Affiliations:** 1 College of Resources and Environment, University of Chinese Academy of Sciences, 19 A Yuquan Road, Shijingshan District, Beijing, 100049 China; 2 Ministry of Agriculture and Rural Affairs, Agro-Environmental Protection Institute, Tianjing, 300191 China

**Keywords:** ARG, Heavy metals, Co-contamination, Sub-MIC

## Abstract

An environment co-contaminated with metals and antibiotics ultimately exposes bacteria to these metals and antibiotics simultaneously. This study aims to explore the efficacy of sublethal concentrations of copper ions contaminated with tetracycline regarding antibiotic resistance in a sensitive strain of *E. coli* K12. The study proved that a copper ions and tetracycline co-contaminated environment could considerably enhance the mutation frequencies of chloramphenicol and polymyxin B resistance in antibiotic susceptible *E. coli*; however, the equivalent copper ions and tetracycline alone showed weaker effects. Results also demonstrated that an environment co-contaminated with relatively high sublethal concentrations of copper ion and tetracycline co-contaminated environment could induce much higher antibiotic resistance than the low sublethal and control groups. Whole-genome characterization results indicated that variability existed within the genotype and phenotype involved in antibiotic resistance. Additionally, the evolved resistant strains displayed hereditary resistance after 5 round culture cycles in LB broth over 5 days. Results implied that co-contamination with metals and antibiotics environment could strengthen resistance and contribute to the induction and dissemination of antibiotic resistance in metal and antibiotic co-contaminated environment.

## Key points

- Mutation frequencies increased with cross contamination.
- Antibiotic resistance induction was dose dependent.
- Genotype and phenotype could diverge under the same stress condition.
- The resistant mutants displayed hereditary resistance.

## Introduction

Antibiotics are the essential double-edged sword: one hand, antibiotics are a powerful weapon to life-saving, on the other hand, misuse and overuse urge bacteria development and dissemination of resistance (Zhu et al. 2013; Lv et al. 2014; Vikesland et al. 2017). Antibiotic resistance forms an increasing risk to our body health, since this is relevant to the therapeutic effects of antibiotics (Vikesland et al. 2017). As the result of over-use of antibiotics induced the selective stress in our environment, more and more antibiotic resistant bacteria (ARB) and antibiotic resistance genes (ARGs) were determined in clinical as well as nature (Alonso et al. 2001). Residual antibiotics from human and animal feces/wastes, hospital waste and pharmaceutical industries eventually contaminate the soil and water environments (Pruden et al. 2006; Nolivos et al. 2019; Povolo and Ackermann 2019). Soil fertilizer with animal waste and sewage sludge are causing antibiotic accumulation (Zhu et al. 2013). Residual antibiotics could run away from the soil environment, then get to the water ecosystem (Martinez 2008). Additionally, antibiotics are regarded as “pseudo-persistent”, although most antibiotics have relatively short half-lives, their residues persist in the environment (Pan et al. 2011). Even at several 100-fold below the minimal inhibitory concentration of antibiotics could enrich antibiotic resistant bacteria (Gullberg et al. 2011).

However, heavy metals are persistent in nature, gathering in a diverse constituent of the ecosystems (Lu et al. 2014). Copper and zinc are generally employed as food additive in animal feed diets through the supplement of the ingredients in their compound feed, exceeding the needs for normal growth of the animals, and for precaution of animal disease, as well as for the growth promotion and medical remedies (Baker-Austin et al. 2006; Hau et al. 2017). Reports claimed that copper in the soil, not only selects for copper resistance but also co-selects for resistance to antibiotics, for example, chloramphenicol, tetracycline and ampicillin (Berg et al. 2005). Most of researches related to antibiotic resistance were focused on high concentrations, such as, more than the minimal inhibitory concentration (MIC)) (Seiler and Berendonk 2012), while the effect of relatively low concentrations, that is sublethal (< MIC) still mainly unclear.

Additionally, sub-inhibitory concentration of metals could endow bacteria antibiotic resistance through co-selection, as well as heavy metal resistance (Gullberg et al. 2014; Chen et al. 2015). Sublethal concentrations of antibiotics induce mutagenesis by stimulating the production of ROS, these effects can result in mutant bacterial that are sensitive to the applied antibiotic but resistant to other antibiotics (Kohanski et al. 2010). As we know, heavy metal pollution in environments could contribute to the maintenance and dissemination of antibiotic resistance (Stepanauskas et al. 2005; Henriques et al. 2016; Zhang et al. 2018a; Imran et al. 2019). Such as, Zhu et al. (2013) reported a significant correlation between copper concentration and the incidence of ARGs (Zhu et al. 2013). In some natural ecosystems, heavy metal and antibiotic co-contaminated together could drive the dissemination of antibiotic resistance in bacteria (Baker-Austin et al. 2006; Wang et al. 2014), and co-exposure to zinc and antibiotic co-contaminated environments such as oxytetracycline in activated sludge bioreactors could enhance the resistance of the microbial community towards antibiotics (Peltier et al. 2010). Findings suggest that the low levels of antibiotics and heavy metals present in polluted external environments could allow for selection and enrichment of bacteria with multi-resistance plasmids (Gullberg et al. 2014). But how sub-inhibitory concentration of tetracycline and copper ion contribute to the emergence and maintenance of clinically significant *E. coli* is largely unknow.

Evolutionary experiments aim to determine whether sublethal concentrations of copper ions contaminated with tetracycline could increase the bacterial antibiotic resistance through stimulation in a co-contaminated environment. Whole-genome sequencing analysis will be utilized to gain insight into the potential mechanisms of resistance. Comparative molecular analysis of resistance determinants, coupled with phenotypic analysis, may contribute to further understanding of antibiotic resistance sources. This study’s findings will clarify the complicated relationship between heavy metal and antibiotic co-contamination with antibiotic resistance. A summary of the changes in resistance with generations on the evolution cycle in *E. coli* will be examined in a further study.

## Materials and methods

### Strains, antibiotics and selection condition

First, *E. coli* K12 (MG1655) was been sequenced (Shanghai Majorbio Bio-pharm Technology Co., Ltd), which was designated as the original wild type strain (Table 1). Activating *E. coli* from storage tube with glycerol stock which stored in − 80 °C, expanding propagating on a Luria Bertani (LB) agar plates, cultured at 37 °C for 16 h. The selected seed strain was cultured in LB broth at 37 °C for 12 h for following selected experiments (Li et al. 2016).

**Table 1:** The MIC of each antibiotic to wild-type *E. coli* K12

No	Antibiotics	Abbreviation	Classification	Stock solution (mg/L)	MIC (mg/L)
1	Ciprofloxacin	Cip	Quinolones	200	0.2
2	Tetracycline	Tet	Tetracyclines	10	2.34
3	Gentamicin	Gen	Aminoglycosides	10	8.75
4	Polymyxin B	Pol	Polypeptides	10	0.94
5	Erythromycin	Ery	Macrolides	6.4	15
6	Chloramphenicol	Chl	Chloramphenicols	30	4.69

The involved antibiotics: chloramphenicol (Chl), ciprofloxacin (Cip), erythromycin (Ery), gentamycin (Gen), tetracycline (Tet), and polymyxin B (Pol) and cupric (CuSO4·5H2O) were get from Solarbio, Inc. (Shanghai, China). 90% inhibition of growth was regarded as the MIC of each antibiotic, which was determined as Additional file 1: Test S1 described, and the MIC of copper ions was also investigated with the same method. The detailed accounts are displayed in Additional file 1: Text S1. The tetracycline resistant cultures were kept from light so as not to degrade the antibiotic.

### Determination of minimum inhibitory concentrations

The MICs were investigated after 40 sub-culture cycles, using previously described methods (Lv et al. 2014). The serially diluted strain cultures were grown on LB agar plates for 16 h at 37 °C. Then 10 colonies from each parallel agar plate were unintentionally selected from each sub-culture. These randomly picked strains were cultured in 3 mL of LB broth for 5–6 h at 37 °C to investigate the MICs (Additional file 1: Text S1) (Li et al. 2016). And, the MICs of the original *E. coli* K12 strain was also determined (Table 1).

### Exposure to copper ions and tetracycline environment

**Fig. 1:**
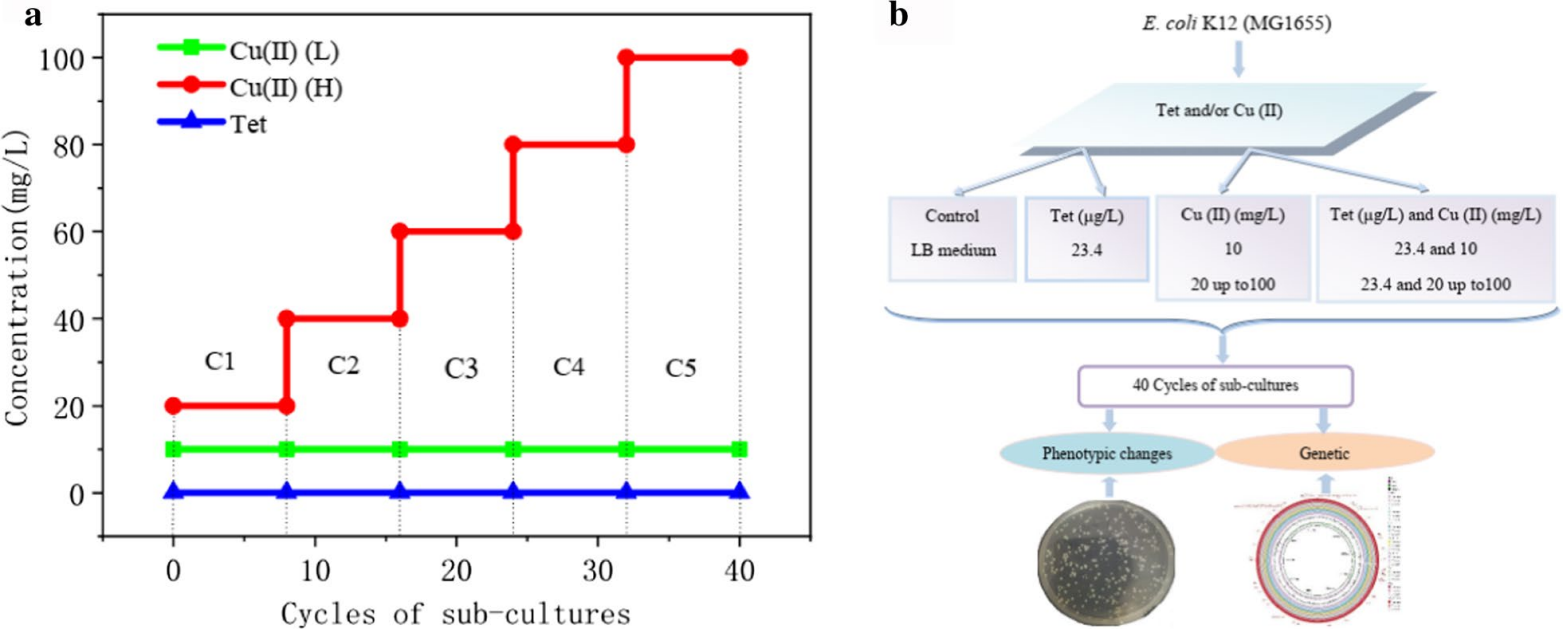
Diagram exposure sub-culture and schematic experimental design. Antibiotic resistance selection by both copper ion (Cu (II)) and tetracycline (Tet). The initial bacterial culture with copper ions and tetracycline treatments or without any copper ions or tetracycline treatment (control groups) as cycle 0 (recorded as C0), and subsequent were recorded as C1, C2, etc. Each cycle was 8 days, a total of 5 cycles. **a** Cycles of the exposure sub-culture; **b** The concentrations of Cu (II) and Tet, during 5 cycles of sub-cultures for antibiotic resistance selection with mental ions of Cu (II) and Tet. The concentration of tetracycline during the experiments was set as 0.0234 mg/L, gradually increasing the copper ion dose concentrations from 1/100 × MIC (20 mg/L) up to 1/10 × MIC (100 mg/L), as the relatively high concentration evolution condition; and relatively lower concentrations of copper ion at about 10 mg/L. H: Strains exposure at high copper ion concentrations; L: Strains exposure at low copper ion concentrations. All experiments were performed in triplicate

Detailed procedures of the exposure experiments are shown in Fig. 1. A sample, totaling 5 mL, including 0.5 mL of original *E. coli* cultures (10^6^ CFU) and 4.5 mL of fresh LB broth, containing corresponding concentrations of copper ions and tetracycline, was cultured in a 15 mL sterilized tube at 37 °C for 24 h. Subsequently, 0.5 mL of the cultures was transferred into a new 15 mL sterilized tube with 4.5 mL fresh LB broth to sub-culture with corresponding copper ions and tetracycline. The original isogenic K 12 was cultured in LB broth; this lacked any copper ions or tetracycline and was used as the control. All experiments were conducted in triplicate.

The exposure dosages of copper ions and tetracycline applied in this research were calculated according to the MICs as well as the concentration of environments (Additional file 1: Fig. S1, Table 1); taking into account the tested and evaluated environmental concentrations (Zhu et al. 2013), examples of which include the cupric concentration in Dawu River, which ranged from 12 to 30 mg/L (Huang Changgan et al. 2004), also the cupric concentration in previous studies which ranged from 8 to 500 mg/L (Chen et al. 2015; Poole 2017). Resistant bacteria can be selected at the high concentrations of tetracyclines used therapeutically, but what role the extremely low tetracycline concentration present plays in selection still largely unclear (Gullberg et al. 2011). Thus, the concentration of tetracycline during the experiments was set as 0.0234 mg/L. The resistance evolution experiments were observed in 5 conditions (Fig. 1b), gradually increasing the dose concentrations from 1/100 × MIC (20 mg/L) up to 1/10 × MIC (100 mg/L), as the relatively high concentration evolution condition; and relatively lower concentrations of copper ions at about 10 mg/L. In the relatively high concentration evolution selection culture, the copper ion concentrations gradually increased from a starting point of 20 mg/L to approximately 100 mg/L (recorded as H) (Fig. 1a). In the relatively low concentration evolution cultures, the concentrations of copper ions and tetracycline were kept consistent at 10 mg/L and 0.0234 mg/L, respectively (recorded as L). All experiments were repeated in triplicate.

The initial bacterial culture with copper ions and tetracycline treatments or without any copper ions and tetracycline (control groups) were recorded as C0. The following sub-cultures were devised as cycle 1, cycle 2, etc., recorded as C1, C2, etc., respectively (Fig. 1a). Four milliliter of every experimental cycle sub-culture was mixed with glycerol solution in a 15% (v/v), then stored at − 80 °C for the subsequent step (Li et al. 2019). All culture steps were incubated with 200 rpm shaking in aerated incubators; each cycle was 24 h, a total of 40 sub-culture cycles were carried out (Fig. 1a).

### Subsequent antibiotic resistance determination

After 40 cycles of sub-culture, the mutation rates were investigated (Kohanski et al. 2010). To determine the antibiotic resistant mutation rates, sub-cultured strains were streaked on LB agar plates containing corresponding antibiotics (Table 1), following cultured for 48 h at 37 °C (Li et al. 2016), and the colonies were counted. The colonies grew on LB agar without any antibiotics were regarded as the total bacterial concentrations. The colonies grew on LB agar plates containing antibiotic were also determined as resistance to the corresponding antibiotics (Lv et al. 2014). The maximum-likelihood method was used to investigate the mutation frequency as previous described (Lv et al. 2014), as the follow formula (1).

1 $${\text{Mutation rates}} = \frac{{\text{Resistant clones}}}{{\text{Total number of clones}}}$$

Fold changes of mutation rate were measured through each exposure treatment relative to an untreated control group.

### Hereditary stability determination

Hereditary stability of the randomly selected clones after 40 cycles was tested for 5 days of sub-culture cycles (Lv et al. 2014; Zhang et al. 2018b). That is, the selected strains were diluted 1:100 in 5 mL of fresh LB media without any treatment, then regrowth for 24 h at 37 °C, 180 rpm. Each selected strain was exposed to 5 such cycles of growth (Zhang et al. 2018b). The MIC values were measured after 5 days of sub-culture, then matched the MICs of the initial strains to determine the hereditary stability. The MIC determination method was used as former research described (Li et al. 2016; Khan et al. 2017) (Additional file 1: Text S1).

### DNA extraction and whole-genome sequencing

The strains from each treated group and the control group cultured 2 to 3 times on LB agar plates without any antibiotics for 16 h at 37 °C. The Universal Genomic DNA Extraction Kit (Takara, Beijing, China) was used to extract the total DNA, the manipulation process was depending on the manufacturer’s instructions. The concentration and purity were investigated by Nanodrop 2000 (Thermo Fisher Scientific, Wilmington, DE).

The NEXTflex™ Rapid DNA-Seq Kit was applied to set up Illumina sequencing libraries. Briefly speaking, 5′ prime ends were first end-repaired and phosphorylated. Next, the 3′ ends were A-tailed and ligated to sequencing adapters. The third step was to enrich the adapters-ligated products using PCR (Shanghai Majorbio Bio-pharm Technology Co., Ltd).

### Data analysis

Microsoft Excel 2016 (Microsoft Inc., USA) was used to manipulate the mean, standard deviation and fold change of data. Independent sample t-test was used to analysis significant differences (SPSS 18.0, USA).

## Results

### Copper ion and tetracycline co-contaminations enhance the mutation frequency of polymyxin B and chloramphenicol

**Fig. 2:**
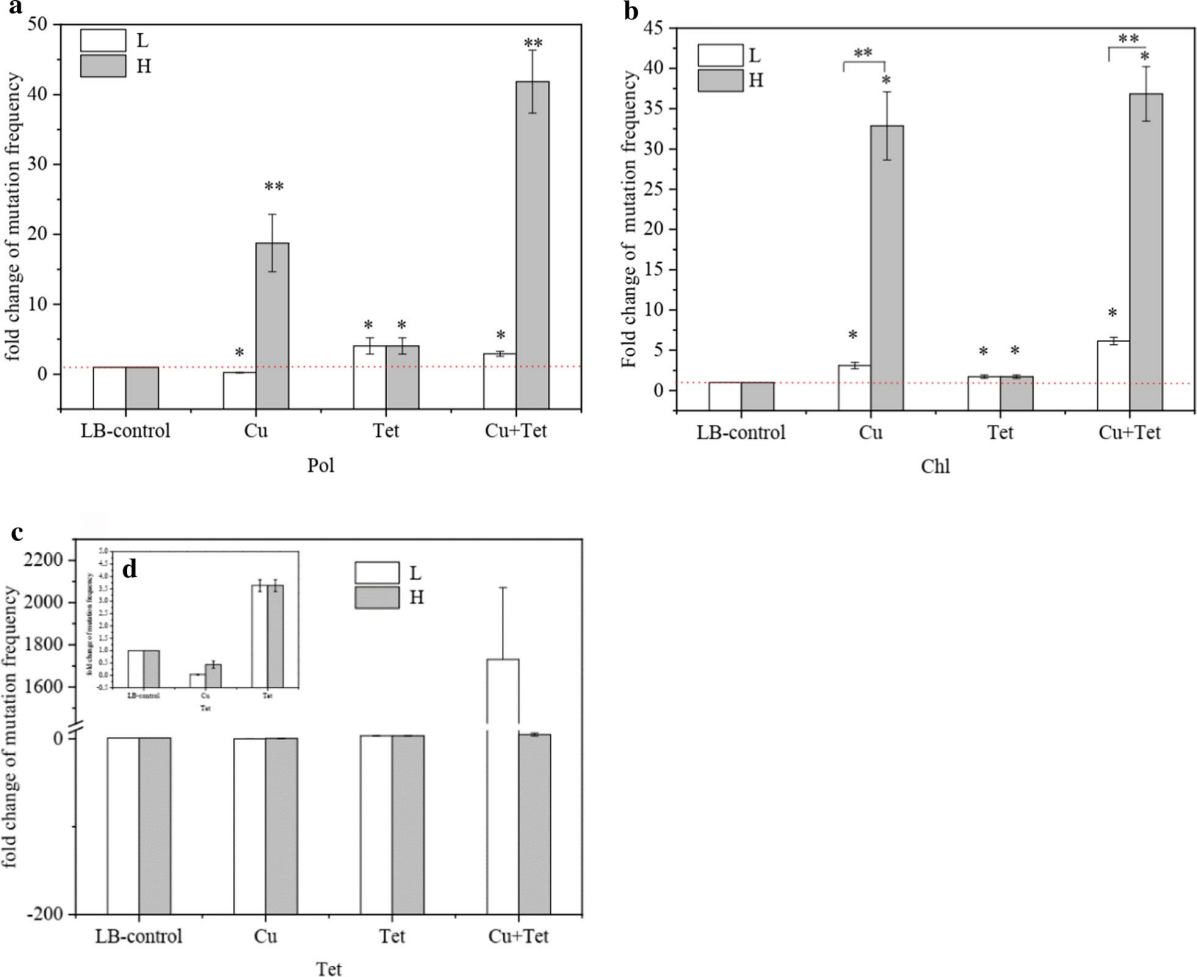
Mutation frequency changes for **a** polymyxin B (Pol), **b** chloramphenicol (Chl) and **c**, **d** tetracycline (Tet) respectively induced by copper ions (Cu) or tetracycline (Tet) and their co-contaminations. LB-control was without any treatment. H: Strains exposure at high copper ion concentrations; L: Strains exposure at low copper ion concentrations. All experiments were performed in triplicate. All experiments were manipulated in triplicate. Significant differences were tested: * (P < 0.05), ** (P < 0.01) and *** (P < 0.001)

Figure 2 indicates the effects on bacterial antibiotic resistance after exposing the *E. coli* strains to the sublethal concentrations of copper ion and tetracycline. The mutation rate of the control, which was not treated by sublethal concentrations of copper ion and tetracycline, was regarded as the spontaneous mutation rate, which ranged from 10^−8^ to 10^−6^ for different antibiotics.

Sublethal concentrations of copper ions and tetracycline co-contaminated treatments could critically enhance the resistant mutation rates of polymyxin B and chloramphenicol (P < 0.05) (Fig. 2a, b), compared with the control groups. The increased mutation rate of resistance to ciprofloxacin and gentamicin was < twofold compared to the control groups (data is not displayed). Sublethal concentrations of copper ions and tetracycline co-contamination resulted in copper dose-dependent alteration in resistance mutation rates for polymyxin B (Fig. 2a) and chloramphenicol (Fig. 2b). The higher the level of copper concentration in the compounds, the higher chance of polymyxin B or chloramphenicol resistance; copper ion treatment alone was similar.

The resistance of *E. coli* K12 to polymyxin B or chloramphenicol showed that relatively high sublethal concentrations of copper ion, acting alone or in combination with antibiotics, can increase the frequency of mutations by 0.2–2.9, 18.8–41.9, 3.1–6.1, and 32.9–36.8 folds respectively, compared with the controls. However, only treatment with a relatively low sublethal concentration of copper ion and tetracycline co-contamination caused a significant increase in the mutation rates for tetracycline resistance. The increase was 1730.8-fold; however, there was only a fivefold increase with a relatively high sublethal concentration of copper ion and tetracycline co-contamination (Fig. 2c). Additionally, both low and high sublethal copper ion treatment induced a mutation resistance to tetracycline with a 0.04- and 0.44-fold increase, respectively (Fig. 2c). Resistance to tetracycline increased 3.64-fold with tetracycline treatment (Fig. 2d).

### Effects of sublethal concentration of copper ion and tetracycline co-contaminations on antibiotic resistance

**Fig. 3:**
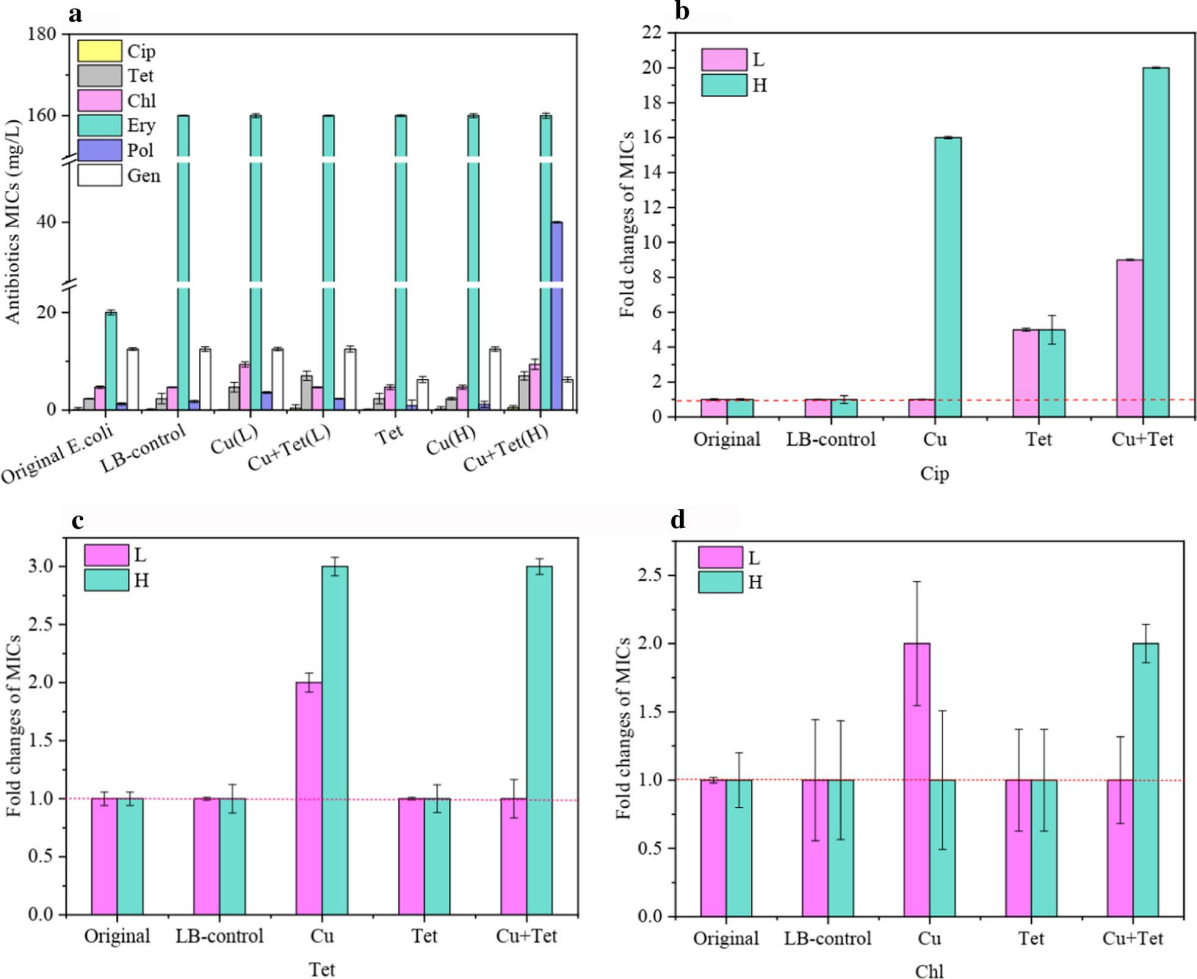
Change of the minimum inhibition concentrations (MICs). **a** Ciprofloxacin (Cip), tetracycline (Tet), chloramphenicol (Chl), gentamicin (Gen), polymyxin B (Pol) and erythromycin (Ery)) among the original *E. coli* K12 in LB medium (LB-control), copper ions and tetracycline alone act or contaminated treatment; fold changes of MICs of ciprofloxacin- (**b**), tetracycline- (**c**), and chloramphenicol-resistant (**d**) induced by copper ion (Cu) and/or tetracycline (Tet) treatment. H: Strains exposure at high copper ion concentrations; L: Strains exposure at low copper ion concentrations

With a sublethal concentration of copper ions and tetracycline exposure, selected strains that demonstrated clinically relevant resistance to ciprofloxacin, erythromycin, polymyxin B, tetracycline, and chloramphenicol with an increase in MICs by 1 to 32-fold (Fig. 3a). The resistance to gentamycin was no more than a onefold increase in MICs (Fig. 3a). The average MICs for environments co-contaminated with sublethal copper ions and tetracycline-selected ciprofloxacin-resistant strains were more than 11–16 and 16–20 folds higher than that of the treatment with copper ion alone or the original or LB-control *E. coli* (Fig. 3b). The relatively high sublethal concentration of copper ion contaminated with tetracycline antibiotic-selected resistant strains displayed over fourfold higher MICs than the relatively low sublethal concentration for ciprofloxacin. Tetracycline-selected ciprofloxacin-resistant strains exhibited fivefold higher MICs than the original or LB-control *E. coli* (Fig. 3b). The tetracycline and chloramphenicol-resistant strains selected with relatively low sublethal copper ion treatment showed higher resistance than the copper ion and tetracycline co-contaminated environment (Fig. 3c, d).

### Whole-genome sequence analysis of the evolution strains

Whole-genome sequencing analysis of the selected mutants was undertaken to determine the underlying genetic mechanisms involved in antibiotic resistance caused by sublethal of copper ions and tetracycline environments. Two or three isogenic clones were selected from the strains tackled by relatively low or high sublethal concentrations of copper ions and tetracycline for whole-genome sequencing analysis (Brockhurst et al. 2011).

**Fig. 4:**
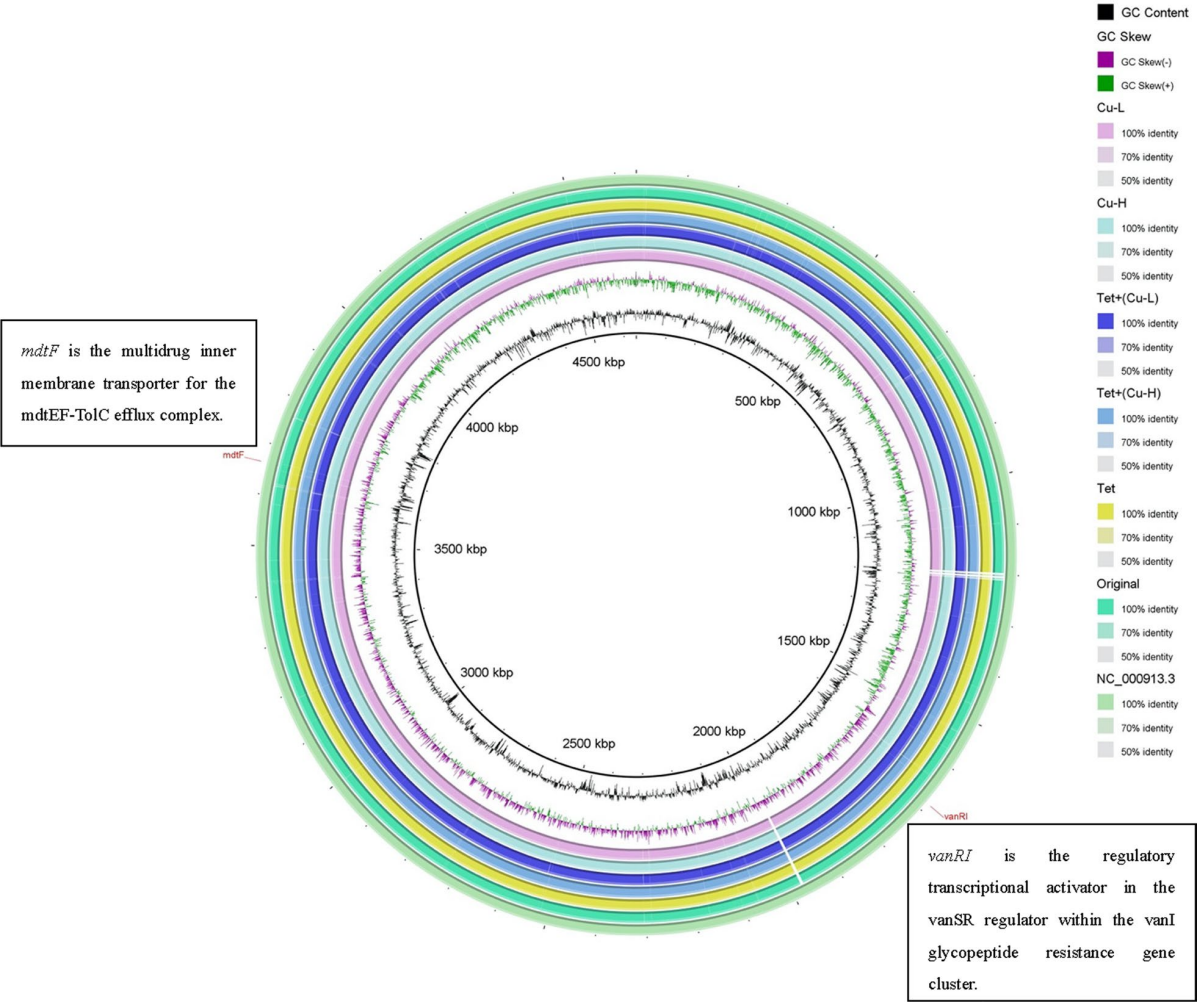
Comparison of genetic changes of genome coverage and mutations identified in strains selected under exposure to copper ion (Cu) and tetracycline (Tet) concentrations, initial *E. coli* K12 and control. Two genetic changes were determined, that is *mdtF* and *vanRI*. The detailed information of mutations on these genetic changes was shown in Table 2

Two genetic changes involved in ARG were determined, that is *mdtF* and *vanRI*, including a substitution mutation and insertion genetic changes induced by copper ion or tetracycline exposure (Fig. 4 and Table 2). Two different changes were also identified on *mdtF* and *vanRI* genes in the LB-control strain, caused by spontaneous mutations throughout the sub-culture processes, such as growing situation and stress (Table 2). One genetic mutation in 1 gene was detected in *E. coli* strains induced by a relatively high sublethal copper ion environment (Fig. 4 and Table 2). The genetic insertion was associated with the membrane transporter gene (*mdtF*) for the mdtEF-TolC efflux complex. Similarly, 1 genetic mutation in 1 gene was detected in *E. coli* strains caused by relatively low sublethal copper ions or tetracycline treatment and relatively low sublethal concentration in their co-contaminated environments; which was associated with a transcriptional activator in the *vanSR* regulator within the *vanI* glycopeptide resistance gene cluster (*vanRI*) (Fig. 4 and Table 2). The number and evolution of clinically relevant antibiotic resistance genes could be induced by contaminations; other genes with main functions, but with a resistance phenotype, are present in the environmental resistome.

**Table 2:** Summary of identified genetic changes in untreated control clones selected in LB medium and in strains selected in exposure environments

Strains names	Site	Types	Gene
Original *E. coli*	No genetic change		
LB-control	1210–1215	TTANGG	*mdtF*
LB-control	572	G → A	*vanRI*
Cu-H	1203–1217	GATAGGGTTANTGGC	*mdtF*
Cu-L	572	G → A	*vanRI*
Tet	572	G → A	*vanRI*
Tet + (Cu-L)	572	G → A	*vanRI*
Tet + (Cu-H)	572	G → A	*vanRI*

The *mdtF* is the multidrug inner membrane transporter for the mdtEF-TolC efflux complex; *vanRI* is the regulatory transcriptional activator in the vanSR regulator within the vanI glycopeptide resistance gene cluster. H: resistant strains selected at high copper ion exposure concentrations that gradually increased up to 1/10 MIC level; L: resistant strains selected at low mental ion exposure concentrations about 1/100 MIC

### The hereditary stability of antibiotic resistance in evolved *E. coli* strains

**Fig. 5:**
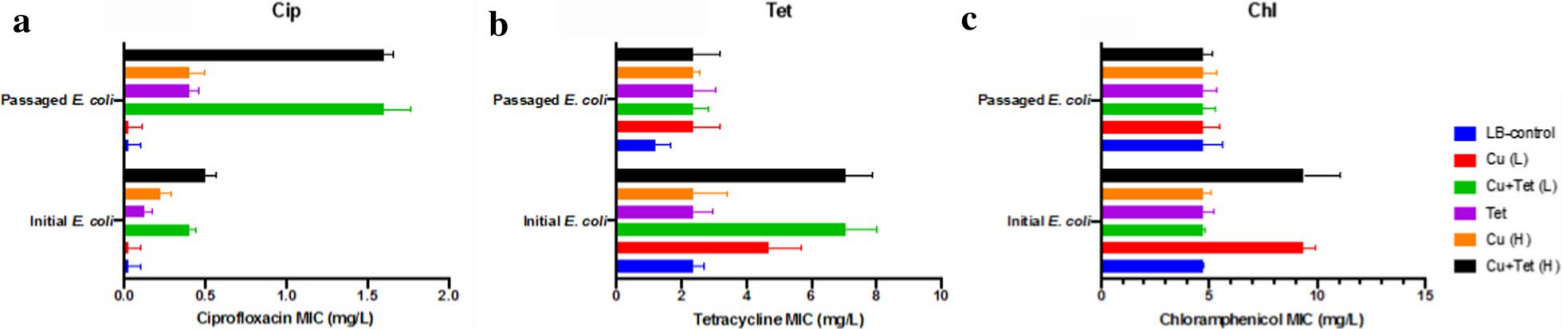
Determination of MICs of **a** ciprofloxacin (Cip), **b** tetracycline (Tet) and **c** chloramphenicol (Chl) for passage cultured *E. coli* K12 with copper ion and tetracycline exposure after 5 days sub-culture. The passaged *E. coli* is derived from the initial *E. coli* K12 (under copper ion and/or tetracycline exposure), which was serially passaged for 5 days sub-culture in LB medium without any copper ions or tetracycline

Hereditary antibiotic resistance in the evolved strains exposure to sublethal concentration of metal and antibiotic co-contaminations is of great significance for human health and environmental safety (Lv et al. 2014; Zhang et al. 2018b). Figure 5 showed ciprofloxacin-, tetracycline-, and chloramphenicol- resistant MICs after 5 culture cycles in LB broth over 5 days. Results indicate that the resistance levels to tetracycline and chloramphenicol were significantly decreased by a relatively low sublethal concentration of copper ion or the relatively high sublethal concentration of the co-contaminations (Fig. 5b, c). Ciprofloxacin’s resistance levels increased significantly with copper ions and tetracycline treatment, except for the relatively low sublethal concentration of copper ion (Fig. 5a).

## Discussions

The present data analysis showed that sublethal concentration of copper ions and tetracycline co-contamination could enrich de novo resistant mutants to increase the corresponding antibiotics’ mutation frequencies. A previous study indicated that environmental pollutions such as metals, biocides, and organometallics could promote the transmission of mobile genetic elements (MGE) through a co-selection mechanism (Kohanski et al. 2010), thereby contributing to antibiotic resistance, our research results are consistent with previous reports. Deficient concentrations of antibiotics (Kohanski et al. 2010; Gullberg et al. 2011), heavy metals (Li et al. 2019), disinfectants (Kohanski et al. 2010), and disinfection by-products (DBPs) (Lv et al. 2014; Li et al. 2016) could promote bacteria evolving antibiotic resistance were also reported. Therefore, together with previous reports, we hypothesized that the presence of ARB in varied sublethal concentrations of copper ions and tetracycline environments are partially understood through the selective effects of co-contaminated environmental chemicals, and the co-selection of co-contaminations driven *E. coli* evolved antibiotic resistance via co-resistance or cross-resistance.

Additionally, many studies proposed that, environmental pollutions, such as metal, can accelerate ARG dissemination (Kohanski et al. 2010; Zhang et al. 2018a). This study suggests that heavy metals and residual antibiotics could result in long-term stress on antibiotic resistance populations.

The MIC determination was performed to investigate phenotypic evidence of resistance to antibiotics. The MICs were analyzed to evaluate the bacterial resistance. *E. coli* displayed the highest resistance to sublethal concentrations of copper ions and tetracycline co-contaminated treatments. The relatively high sublethal concentration of co-contamination was the most toxic compared with the relatively low sublethal or copper ions or tetracycline alone (Fig. 3). It is clear that the environment in which organisms exist affects their susceptibility to antibiotics, confirmed by the selection and enrichment of antibiotic-resistant organisms without antibiotic exposure (Kohanski et al. 2010). Two different changes were respectively identified on *mdtF* and *vanRI* genes in the LB-control strain, could involve in the sub-culture environment. Common sources of environmental stress include metal ions, especially copper and zinc, which at low concentrations are essential for the function of normal bacterial cells and are necessary for some metalloproteins; however, they are toxic at high levels (Lemire et al. 2013). These metals provide selective stress for metal resistance at more elevated levels, which in turn is driven by genetic and physiological links (Fig. 4). Therefore, sublethal concentrations of copper ions and tetracycline co-contamination resulted in copper dose-dependent alteration in resistance mutation rates for polymyxin B (Fig. 2a) and chloramphenicol (Fig. 2b); the higher the level of copper concentration in the compounds, the higher chance of polymyxin B or chloramphenicol resistance; copper ion treatment alone was similar can be explained. In environments contaminated with copper and tetracycline, the enrichment of antibiotic-resistant organisms may be the selection of organisms on chromosomes or plasmids that carry resistance genes for both drugs (Table 2 and Fig. 4).

Previous reports suggested that chromosomal mutation is the primary way and mechanism for bacteria to acquire ARGs, such as base substitution or frameshift in specific genes (Kohanski et al. 2010). Several research reports suggest that sublethal concentrations (far below the MIC) of antibiotics can also induce mutation rate increases for bacteria, for example, in our study, sublethal concentrations of copper ions and tetracycline co-contaminated treatments critically enhance the resistant mutation rates of polymyxin B and chloramphenicol (P < 0.05) (Fig. 2a, b). Furthermore, these mutations appear at a high frequency due to the relatively low fitness-cost (Gullberg et al. 2011). This phenomenon indicated that the sublethal concentration of antibiotics is adequate at acquiring ARGs. Hence, this may explain that with a sublethal concentration of copper ions and tetracycline exposure, selected strains demonstrated clinically relevant resistance to ciprofloxacin, erythromycin, polymyxin B, tetracycline, and chloramphenicol with a significant increase in MICs.

The *mdtF* is a multi-antibiotic inner membrane transporter for the mdtEF-TolC efflux complex. It is a resistance nodulation division (RND) type efflux pump in *E. coli*, with vital homology to *acrB*, but is generally expressed at a low level in clinical isolates. A previous study indicated that overexpressed pumps could induce susceptibility to erythromycin (Bohnert et al. 2007). All Gram-negative strains have genes for efflux pumps that are the part of the RND family. These pumps effectively develop resistance to multiform complex mixtures, for example, antibiotics, dyes, and other xenobiotics, even multidrug resistance (MDR) (Jellen-Ritter and Kern 2001; Webber et al. 2005). The various MICs of most of the antibiotics were involved in detecting a single point mutation; this single-point mutation increases the resistance to exposed antibiotics but reduces the resistance to another antibiotic (Bohnert et al. 2007). These facts allow us to explain why the phenotype and genotype are inconsistent in this study.

*VanRI* gene is a regulatory activator of transcription in *vanSR* regulators within the *vanI* glycopeptide resistance gene cluster (ARO:3003728), a glycopeptide resistance gene conferring antibiotic resistance via molecular bypass. The *mdtF* and *vanRI* mutations might contribute to developing ciprofloxacin, chloramphenicol, and polymyxin B resistance in *E. coli* (Figs. 2 and 3). The above findings indicate that at the relatively high copper ions sublethal contamination (below 1/10 of the MIC) with tetracycline in polluted environments could increase mutation frequency. In this study, the number and uniqueness of genetic alterations were different from mutants caused by varied sublethal of copper ions and tetracycline co-contaminated environments; phenotypes and genotypes showed significant inconsistency.

The evolution and dissemination of antibiotic resistance are accelerated through the acquiring of ARGs via de novo mutation. Genetic mutations are regarded as significant pathways that contribute to the evolution of antibiotic resistance (Nolivos et al. 2019). The attenuation, persistence, and enrichment of ARGs or ARB in the environment are ecological evolution processes stimulated by diverse circumstances (Andersson and Hughes 2011).

The results of the hereditary stability of antibiotic resistance in evolved *E. coli* strains may be explained by the contribution of genotypic-phenotypic discrepancies (Beaber et al. 2004; Corona and Martinez 2013). Strains subjected to antibiotics, whether by increased or decreased resistance level, could relate to phenotypic changes (not-inheritable resistance) and genetic changes (inheritable resistance). Evolved strains considerably raised ciprofloxacin resistance and matched the initial *E. coli* K12 and the control strains; this may have been obtained via genetic changes, based on the whole-genome sequencing analysis. In terms of the adaptive evolution of resistant strains, the stable hereditary features of co-contaminations-induced ciprofloxacin resistance could pose a potential public health threat. In this study, ciprofloxacin resistance can be stably passed on to offspring by evolved strains; this hereditary stability could cause continued antibiotic resistance development.

This study demonstrates a synergistic effect between sublethal doses of antibiotic and ionic copper, both individually and in combination, on stable mutations that lead to evolution of antibiotic resistant bacteria. Evidence was provided that sublethal concentrations of copper ions and tetracycline co-contaminated treatment could lead to significant increase in the evolution of polymyxin B and chloramphenicol resistant mutations. With sublethal concentration of copper ions and tetracycline exposure, the MICs of selected strains resistance to ciprofloxacin, erythromycin, polymyxin B, tetracycline and chloramphenicol increase from 1 to 32 folds. In addition, two genetic changes involved in the antibiotic resistance gene were determined, that is *mdtF* and *vanRI*. Hereditary antibiotic resistance of the evolved strains showed that the evolved resistance can be lost over subsequent generations, though not always. These results might contribute to understand the emergence and dissemination of antibiotic resistance in our environment.

## Supplementary Information

**Additional file 1: Text S1.** Minimum inhibitory concentrations (MICs) determination.** Fig S1.** The MIC of Copper ions to wild-type *E. coli* K12.
