# Supplementary material for: Acceleration of emergence of E. coli antibiotic resistance in a simulated sublethal concentration of copper and tetracycline co-contaminated environment
Source: AMB Express. 2021 Jan 7;11:14. doi: 10.1186/s13568-020-01173-6 (PMC7790946; doi:10.1186/s13568-020-01173-6)
Supplement: Supplementary file 1 — Additional file 1: Text S1. Minimum inhibitory concentrations (MICs) determination. Fig S1. The MIC of Copper ions to wild-type E. coli K12. [file 13568_2020_1173_MOESM1_ESM.docx]

Additional file

**AMB Express**

**Acceleration of emergence of *E. coli* antibiotic resistance in a simulated sublethal concentration of copper and tetracycline co-contaminated environment**

Jinmei Li^a^, Irfan Ali Phulpoto^a^, Guilong Zhang ^b^, Zhisheng Yu^a*^

*^a^* College of Resources and Environment, University of Chinese Academy of Sciences, 19 A Yuquan Road, Shijingshan District, Beijing 100049, China

*^b^* Agro-Environmental Protection Institute, Ministry of Agriculture and Rural Affairs, Tianjing, 300191, China

***Corresponding author:**  Zhisheng Yu

College of Resources and Environment

University of Chinese Academy of Sciences

19 A Yuquan Road, Shijingshan District, Beijing 100049, China

Tel: + 86 10 88256057

Email: yuzs@ucas.ac.cn

**Caption list**

**Text S1 Minimum inhibitory concentrations (MICs) determination**

**Fig S1.** **The MIC of Copper ions to wild-type *E. coli* K12**

**Reference**

**Text S1** **Minimum inhibitory concentrations (MICs) determination**

The MICs were determined as previous described methods (Wiegand et al., 2008). In brief, the overnight culture of *E. coli* K12 was diluted to the density below 0.05 (about 10^6^ CFU/mL) at 600 nm was measured by a microplate reader (Synergy MDMulti-Mode, Cisbio), serial twofold dilutions were introduced into 96-well microplates, and cultured at 37 °C for 24 h, then using microplate reader (Synergy MDMulti-Mode, Cisbio) measured the OD600 (600 nm). Sterilized PBS (pH: 7.1) was considered as blank control. The concentrations of antibiotics or copper ion of *E.coli* K12 that resulted in 90 % growth inhibition of the original *E.coli* cells was considered as the MICs (Li et al., 2016). Each test was performed at least in triplicate.

**Fig S1.** **The MIC of Copper ions to wild-type *E. coli* K12**

**Fig. S1.** The concentration of MIC of copper ions is 1000mg/L. The dose-depended growth inhibitory curves of *E. coli* with the treatment of various concentrations of copper ion. The concentrations of copper ion of *E. coli* K12 that caused 90 % growth inhibition of the original *E. coli* cells was regarded as the MICs. All manipulations were performed in triplicate, and error bars represent ± SD (standard deviation).

**Reference**

Li D, Zeng S, He M, Gu A Z (2016) Water Disinfection Byproducts Induce Antibiotic Resistance-Role of Environmental Pollutants in Resistance Phenomena. Environ Sci Technol 50: 3193-3201

Wiegand I, Hilpert K, Hancock R E (2008) Agar and broth dilution methods to determine the minimal inhibitory concentration (MIC) of antimicrobial substances. Nat Protoc 3: 163-175
